# Anti-PCSK9 Treatment Attenuates Liver Fibrosis via Inhibiting Hypoxia-Induced Autophagy in Hepatocytes

**DOI:** 10.1007/s10753-023-01865-8

**Published:** 2023-07-19

**Authors:** Liuxin Ning, Yanting Zou, Shuyu Li, Yue Cao, Beili Xu, Shuncai Zhang, Yu Cai

**Affiliations:** 1 Department of Gastroenterology and Hepatology, Zhongshan Hospital, Fudan University, Shanghai, 200032 China; 2 Shanghai Institute of Liver Diseases, Shanghai, 200032 China

**Keywords:** Hypoxia, Autophagy, Proprotein convertase subtilisin/kexin type 9, AMPK, Liver fibrosis

## Abstract

Hypoxia and its induced autophagy are involved in the initiation and progression of liver fibrosis. Proprotein convertase subtilisin/kexin type 9 (PCSK9) has been recognized as a potential regulator of autophagy. Our previously reported study found that PCSK9 expression increased in liver fibrosis and that anti-PCSK9 treatment alleviated liver injury. This study aimed to investigate the mechanism of anti-PCSK9 treatment on liver fibrosis by inhibiting hypoxia-induced autophagy. Carbon tetrachloride-induced mouse liver fibrosis and mouse hepatocyte line AML12, cultured under the hypoxic condition, were established to undergo PCSK9 inhibition. The degree of liver fibrosis was shown with histological staining. The reactive oxygen species (ROS) generation was detected by flow cytometry. The expression of PCSK9, hypoxia-inducible factor-1α (HIF-1α), and autophagy-related proteins was examined using Western blot. The autophagic flux was assessed under immunofluorescence and transmission electron microscope. The mouse liver samples were investigated via RNA-sequencing to explore the underlying signaling pathway. The results showed that PCSK9 expression was upregulated with the development of liver fibrosis, which was accompanied by enhanced autophagy. In vitro data verified that PCSK9 increased via hypoxia and inflammation, accompanied by the hypoxia-induced autophagy increased. Then, the validation was acquired of the bidirectional interaction of hypoxia-ROS and PCSK9. The hypoxia reversal attenuated PCSK9 expression and autophagy. Additionally, anti-PCSK9 treatment alleviated liver inflammation and fibrosis, reducing hypoxia and autophagy in vivo. In mechanism, the AMPK/mTOR/ULK1 signaling pathway was identified as a target for anti-PCSK9 therapy. In conclusion, anti-PCSK9 treatment could alleviate liver inflammation and fibrosis by regulating AMPK/mTOR/ULK1 signaling pathway to reduce hypoxia-induced autophagy in hepatocytes.

**Supplementary Information:**

The online version contains supplementary material available at 10.1007/s10753-023-01865-8.

## INTRODUCTION

Hypoxia, known to be involved in the whole process of liver fibrosis, causes the microenvironmental homeostasis imbalance due to the alterations in cellular metabolism, and the oxidative stress-related autophagy induced by hypoxia is a major contributor to the development of liver fibrosis [1, 2]. Autophagy performs complex regulatory functions in different cellular contexts [3]. It is widely recognized that autophagy in hepatocytes could degrade intracellular abnormal proteins and reduce the expression of pro-inflammatory cytokines, thereby alleviating liver fibrosis. On the other hand, autophagy in hepatic stellate cells (HSCs) could promote liver fibrosis by providing energy for the activation of HSCs [4]. Although much has been done on anti-hepatic fibrosis by inhibiting HSC autophagy in previously reported studies, little progress has been made there. Since hepatocytes account for more than 80% of the liver tissue and function as the main functional cells responsible for liver metabolism and detoxification, hypoxia-induced autophagy mainly occurs in hepatocytes in the initial stage. The change of autophagic flux can deliver inflammatory signals to the liver microenvironment, leading to the progression of liver fibrosis. Therefore, there potentially exists a novel strategy for exploring the mechanism of hypoxia-induced autophagy in hepatocytes so that an efficient and precise regulatory approach can be developed to treat liver fibrosis clinically.

PCSK9, a serine protease, is mainly secreted by hepatocytes binding to low-density lipoprotein receptor (LDLR) and promoting its internalization and lysosomal degradation [5]. Thus, PCSK9 inhibition increases LDLR expression in hepatocytes, enhancing circulating LDL uptake and reducing plasma cholesterol levels. PCSK9 monoclonal antibody (mAb) that inhibits the combination of PCSK9 and LDLR has been widely used in the treatment of cardiovascular diseases with abnormal lipid metabolism, with no serious adverse reactions seen clinically [6]. Nevertheless, the role of anti-PCSK9 treatment is not limited to LDL-cholesterol regulation [7]. PCSK9 inhibitor plays an important role in the clearance of pathogenic lipids including lipopolysaccharide (LPS). Several lines of evidence have suggested that anti-PCSK9 therapy could positively affect sepsis and septic shock [8–10]. In our previously reported study, the evidence showed that anti-PCSK9 treatment ameliorated liver injury by enhancing LPS uptake in hepatocytes [11]. Additionally, PCSK9 is known to be involved in the regulation of autophagy, playing different roles in a variety of cell types. In the primary mouse cardiomyocytes, PCSK9 inhibition could significantly reduce autophagy via activation of the ROS-ATM-LKB1-AMPK signaling pathway [12]. Furthermore, anti-PCSK9 therapy has a promising potential of reducing autophagy by inhibiting mammalian rapamycin targeting protein (mTOR) via increasing protein kinase B. However, the role of anti-PCSK9 therapy remains controversial in treating liver fibrosis, liver inflammation, and nonalcoholic steatohepatitis (NASH) [13–15].

In the current study, we discovered the effect of anti-PCSK9 therapy on liver fibrosis when the hypoxia-induced autophagy was inhibited in hepatocytes. In investigating the probable signaling pathway involved in anti-PCSK9 treatment, furthermore, we observed the experimental evidence that anti-PCSK9 therapy could be an effective anti-fibrosis agent.

## MATERIALS AND METHODS

### Reagents

A list was made of the reagents (Supplementary Table S1).

### Cells

The mouse liver cell line AML12 (Zhong Qiao Xin Zhou Biotechnology Co., Ltd., Shanghai, China) were cultured in DMEM/F12 supplemented with 10% fetal bovine serum (FBS) and 1% penicillin/streptomycin (P/S). The mouse mononuclear macrophage cell line RAW264.7 were purchased from National Collection of Authenticated Cell Cultures and cultured in DMEM supplemented with 10% FBS and 1% P/S. The cells were maintained under a humidified atmosphere of 95% air and 5% CO_2_ in an incubator at 37 °C. The hypoxic condition was created by incubating hepatocytes in a controlled atmosphere incubator with an atmosphere of 1% O_2_, 5% CO_2_, and balance N_2_ at 37 °C. The cells, seeded in cell culture flasks with serum-containing medium, were routinely passaged every 3 days using trypsin-ethylenediaminetetraacetic acid. All incubations were performed under four passages.

Primary hepatocytes were routinely isolated from male C57BL/6 J mice through in situ enzymatic digestion of the liver with the collagenase I method, followed by density centrifugation, as previously described [16]. The freshly isolated hepatocytes were seeded into collagen-coated Petri dishes to be grown in RPMI medium 1640 supplemented with 1.0 g/L glucose, 10% FBS, and 1% P/S in a humidified atmosphere of 5% CO_2_ at 37 °C. The medium was changed 6 h after plating. PCSK9 inhibitor Evolocumab (Amgen Inc., USA) at a concentration of 100 μg/mL was then incubated for 24 h in hepatocytes.

### Cell Transfection

AML12 cells were seeded into 6-well culture plates 24 h before transfection. Reaching 70–80% confluence, the cells were separated into four groups to be transfected with each of the following siRNAs: non-targeting scrambled control siRNA (siNC) group, siRNA1 group, siRNA2 group, and siRNA3 group. AML12 cells were transfected with PCSK9 siRNA and HIF-1α siRNA, respectively. These reagents were transfected into cells via Lipo8000 Transfection Reagent according to the manufacturer’s protocol. The medium was changed 6 h after transfection. Following siRNA transfection and 24-h incubation, the follow-up experiments were conducted. A list was made of the synthesized oligos (Supplementary Table S2). Knockdown (KD) efficiency was determined by qRT-PCR and Western blot (Supplementary Figure S1 and Table S3).

### Animals

Male C57BL/6 J mice aged 4–6 weeks, purchased from Slack Laboratory Animal Co., Ltd. (Shanghai, China), were maintained in a light and temperature controlled environment, with free access to water and food. With 1 week of adaptive feeding, the mice were randomly divided into the following 7 groups: negative control (NC), carbon tetrachloride (CCl_4_)-treated, NC + AAV8-sgPCSK9, CCl_4_ + AAV8-sgPCSK9, CCl_4_ + Evolocumab, NC + YC-1, and CCl_4_ + YC-1. In all NC groups, the mice were injected intraperitoneally with olive oil (5 μL/g) twice a week for 8 weeks. In all CCl_4_ groups, the mice were intraperitoneally injected with a reagent consisting of 10% CCl_4_/olive oil (5 μL/g) twice a week. The Evolocumab-treated mice received weekly subcutaneous injections of Evolocumab (10 μg/g), and the AAV8-sgPCSK9-treated mice received CRISPR-Cas9 adeno-associated virus (AAV) via tail vein 2 weeks before intraperitoneal injection of olive oil or CCl_4_. The AAV injection via tail vein was repeated 1 month later. Those which were treated with YC-1 were intraperitoneally injected with YC-1 (10 μg/g) daily. All mice were euthanized 48 h after the last injection of olive oil or CCl_4_. The blood and liver samples were collected for subsequent evaluation. All animal procedures were performed in accordance with the guiding principles for the care and use of laboratory animals approved by the Animal Care Committee of Zhongshan Hospital and Fudan University.

### Elisa for PCSK9 and TNF-α

In the mouse serum samples or cell culture medium, PCSK9 secretion was measured using the mouse PCSK9 ELISA kit. Similarly, in the mouse serum samples or cell culture medium, tumor necrosis factor-α (TNF-α) was done using the mouse TNF-α ELISA kit according to the manufacturer’s instructions. The linear regression was performed with different concentrations of standards. The samples were combined with peroxidase-labeled IgG anti-PCSK9 or anti-TNF-α antibody attached to the wells of a 96-well plate, which was followed by enzymatic reaction and substrate supplement to be measured at 450 nm with a microplate reader. In each sample, the concentration was calculated according to the linear equation.

### Western Blot

AML12 cells and liver tissues homogenized in RIPA buffer underwent a 10-min centrifugation at 4 °C for 10,000 × g. The protein concentration was assessed by BCA method. With the samples heated up to 100 °C for 10 min with loading buffer, 20 μg of protein was resolved by electrophoresis on 10–15% SDS-PAGE gels and transferred to cellulose nitrate filtration membranes. The membranes were blocked for 1 h in double distilled water (ddH_2_O) with 5% bovine serum albumin (BSA) powder at room temperature, before incubated overnight at 4 °C with 1:1000 diluted primary antibodies. A list was made of the primary antibodies for Western blot assay (Supplementary Table S4). Then, washed with TBST (100 mM Tris–HCl, pH 7.5, 0.9% NaCl, 0.1% Tween 20), the membranes were incubated for 1 h with goat anti-rabbit or goat anti-mouse secondary antibodies at room temperature. The expressed proteins were measured with ECLTM Western Blotting Detection Reagents, and the images were recorded using ChemiScope 6000 (Qinxiang Scientific Co., Ltd., Shanghai, China). To determine the optical density of the bands, ImageJ software (NIH, Bethesda, MD, USA) was used.

### Immunohistochemistry

When formalin-fixed and paraffin-embedded, the liver tissue sections were sectioned at 4 µm to be deparaffinized, hydrated, and subjected to heat-induced antigen retrieval according to the standard protocols as previously described [17]. The liver sections were blocked to be incubated overnight at 4 °C with anti-PCSK9 antibody (Abcam, ab31762) diluted (1:200) in double distilled water containing 5% BSA. The sections were subsequently washed before incubated with HRP-conjugated goat anti-rabbit IgG secondary antibodies (Jackson; 1:500), followed by incubation with 3, 3′-diaminobenzidine tetrachloride for 5–10 min and visualization of specific staining by light microscopy. Under a high-power field with BX51 (Olympus, Japan), the images were captured.

### Immunofluorescence

After 1-h blocking with 5% BSA at 37 °C, the frozen sections of liver tissue were incubated overnight at 4 °C with HIF-1α antibody (CST, 36169) diluted (1:400) in double distilled water containing 5% BSA and then at 37 °C with Cy3-conjugated goat anti-rabbit IgG (Servicebio, 1:100) for 1 h in the dark. The images were taken under confocal microscopy (FV3000, Olympus, Japan).

### Measurement of ROS by Flow Cytometry

Grown in six-well plates, AML12 cells were harvested after hypoxia treatment or mPCSK9 protein stimulation for 24 h. The quantitative measurements of total cellular ROS generation were performed using a Reactive Oxygen Species Assay Kit. The cells were stained with 10 mM DCFH-DA for 20 min at 37 °C. The cellular ROS levels were detected by flow cytometry (Bioscience Aria III, Becton Dickinson, USA). The data were analyzed on FlowJo software (V10.8.1, Becton Dickinson, USA).

### Transmission Electron Microscope

The mice samples were perfused with Ringer’s solution before undergoing 0.15 M cacodylate buffer containing 2.5% glutaraldehyde, 2% paraformaldehyde, and 2 mM CaCl_2_ at 37 °C for 5 min. With post-perfusion, the liver tissue was carefully excised and fixed again in the same fixative overnight at 4 °C. Washed in 0.15 M cacodylate buffer (3 × 5 min), the tissue was post-fixed in 1% osmium tetroxide and 0.3% potassium ferrocyanide in 0.15 M cacodylate buffer with 2 mM CaCl_2_ for 1 h in the dark, which was followed by 3 × 10 min’ washes in ddH_2_O. The liver tissue was en bloc stained with 2% aqueous uranyl acetate overnight at 4 °C, before dehydrated in a 30%, 50%, 70%, and 100% Spurr’s resin mixed with 100% ethanol, embedded in fresh 100% Spurr’s resin in silicon molds, and polymerized at 60 °C for 48 h. Once polymerized, the resin blocks were faced, with 70 nm ultrathin sections cut on a Leica EM UC7 ultramicrotome (Leica-Microsystems, Vienna, Austria), picked up on copper formvar/carbon support film grids (PN FCF100H-Cu, Electron Microscopy Sciences, Hatfield, PA), and post-stained with 2% uranyl acetate for 15 min, then with Reynolds lead citrate for 2 min, before imaged on a JEOL JEM-1400 TEM with an AMT XR111 8 Megapixel scintillated CCD camera.

### Whole-Transcriptome Amplification and RNA-Sequencing Analysis

Isolated from the liver tissues using Trizol reagent, total RNA was applied to RNA-sequencing (RNA-seq) analysis. Library preparation was performed on an Apollo Library Prep System (Takara, Shiga, Japan) using the TruSeq Stranded mRNA Sample Prep Kit and Liver RNA-seq on an Illumina HiSeq 4000 platform performing the 75-base single-end sequencing. TopHat (v.2.0.13) and hisat2 were used to map the clean reads to each gene, with the raw data normalized to Fragments Per Kilobase of exon model per Million mapped fragments (FPKM) for subsequent analyses. Bioinformatics analyses were performed as previously described [18, 19]. Differentially expressed genes (DEGs) were identified with the limma package, which implemented an empirical Bayesian approach to estimating gene expression changes using the moderated *t*-test. |log FC|> 0.5 and *p* < 0.05 were considered as the cutoff criteria for screening DEGs. The functional enrichment analyses of the detected DEGs were performed with the cluster Profiler package, with the terms of Gene Ontology (GO) and Kyoto Encyclopedia of Genes and Genomes (KEGG) identified with a cutoff of* p* < 0.05.

Furthermore, Gene Set Enrichment Analysis (GSEA) was used to identify the pathways that were upregulated or downregulated in the CCl_4_-treated mice with/without PCSK9 inhibition. From the MSigDB database, the gene sets were obtained for analysis. And the enrichment analyses of DEGs were performed using Metascape. With the pathway discovered, Cytoscape (Ver3.9.1) was employed to search for differential genes. With all this done, the mechanism diagram was rendered on Biorender software.

### Statistical Analysis

All data were presented as mean ± standard deviation (SD). The statistical differences between two groups were determined using the unpaired *t*-test. The multiple comparisons were analyzed through one-way analysis of variance (ANOVA). Statistical analysis was performed using Prism version 8.0.2 (GraphPad Software, San Diego, CA), all comparisons considered statistically significant when *p* < 0.05.

## RESULTS

### PCSK9 Expression and Autophagy Parallel in CCl_4_-Induced Liver Fibrosis

**Fig. 1:**
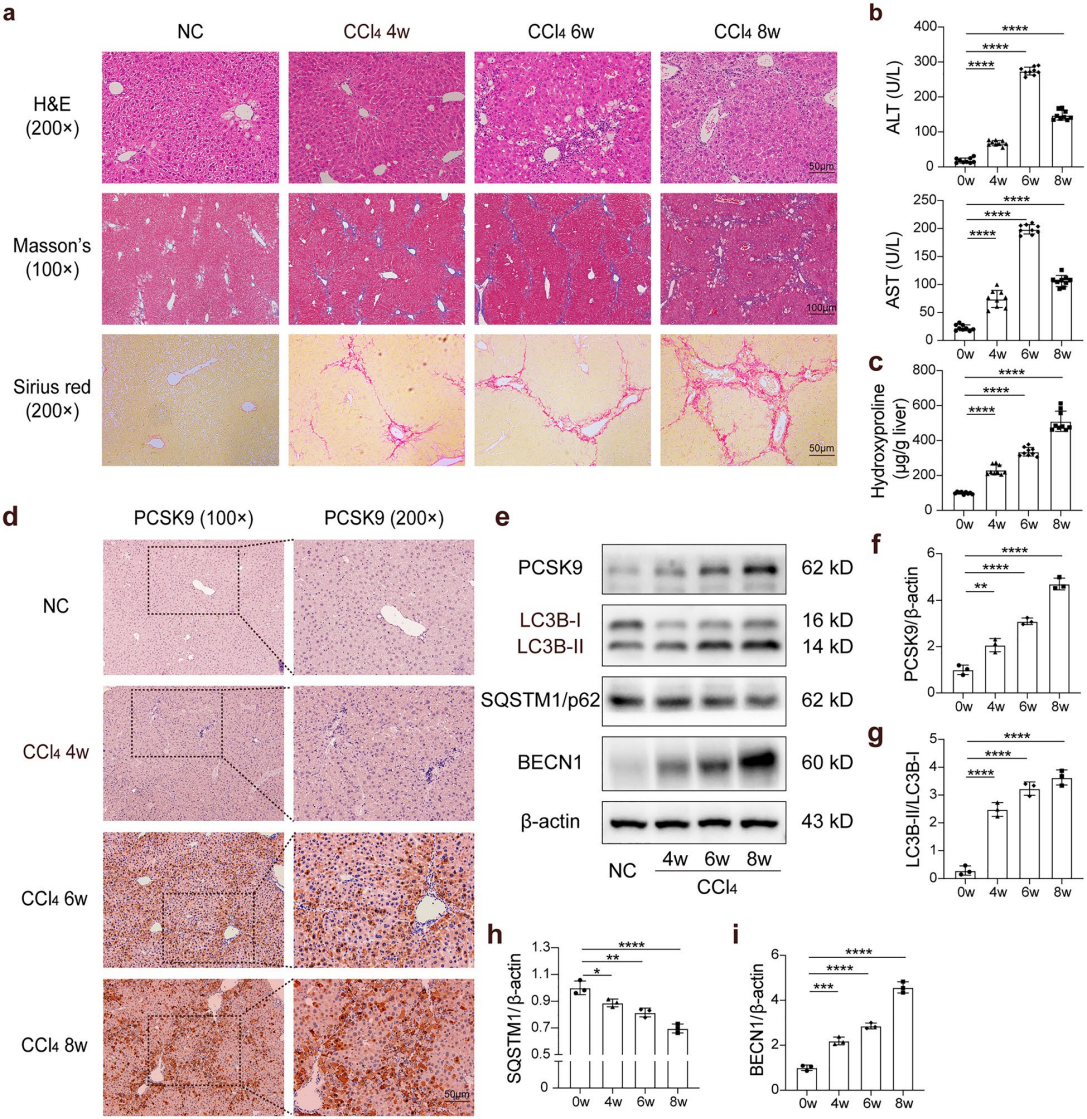
Hepatic function, PCSK9 expression, and autophagy in the CCl_4_-induced liver fibrosis model. **a** H&E, Masson’s, and Sirius red staining of the liver tissue sections treated with CCl_4_ for 4, 6, and 8 weeks. **b** Serum ALT and AST levels. **c** Hydroxyproline content in liver tissue. **d** Immunohistochemical staining of PCSK9 protein in the liver of CCl_4_-treated mice. **e** Western blot of PCSK9, LC3B, SQSTM1/p62, and BECN1 protein after CCl_4_ treatment. **f**–**i** Quantification of relative protein levels calibrated with β-actin; Western blots representative of multiple independent experiments (*n* = 3); multiple comparisons performed by one-way ANOVA; data presented as mean ± standard deviation (*n* = 9 mice/group); *p < 0.05; **p < 0.01; ***p < 0.001; ****p < 0.0001.

In a canonical mouse model of liver fibrosis induced with CCl_4_ for 4, 6, and 8 weeks, respectively, the degree of liver fibrosis aggravated gradually with the extension of CCl_4_ intraperitoneal injection time, the infiltration of the inflammatory cells in H&E staining increased gradually in the liver tissue sections, and the amount of collagen deposition increased, as shown by Masson’s and Sirius red staining (Fig. 1a). According to the serological analysis, the serum levels of alanine transaminase (ALT) and aspartate transaminase (AST) were significantly higher in the CCl_4_ groups than that in the negative control group (Fig. 1b). Serum ALT and AST levels were found to be the highest after 6 weeks of treatment, which was followed by a slight decline 8 weeks later. The hepatic hydroxyproline content experienced a gradual increase after CCl_4_ treatment (Fig. 1c). Evaluated via immunohistochemistry (IHC), the expression of PCSK9 increased gradually with the aggravation of liver fibrosis, concentrated mainly around the fibrotic areas (Fig. 1d). Consistent with the observations, the increased expression of PCSK9 protein was further confirmed using Western blot (Fig. 1e, f).

With autophagy assessed in response to liver fibrosis by measuring the expression of LC3B, SQSTM1/p62, and BECN1 (Fig. 1e), the conversion of LC3B-I to LC3B-II was an essential step in the formation of autophagosomes, and the abundance of LC3B-II correlated with the number of autophagosomes. It was reported that SQSTM1/p62 was a selective substrate for autophagy and that autophagic flux resulted in decreased p62 expression [20]. BECN1 is also a well-known key regulator of autophagy [21]. We observed that autophagic flux was enhanced with increasing degree of liver fibrosis. The expression of LC3B-II/I (Fig. 1g) and BECN1 (Fig. 1i) was maximal, and p62 (Fig. 1h) expression was minimal 8 weeks later after CCl_4_ treatment.

Therefore, the evidence indicated that PCSK9 expression and autophagic activity increased in parallel with the progression of liver fibrosis; hence, there may be a functional interaction between PCSK9 and autophagy in CCl_4_-induced liver fibrosis.

### PCSK9 Increased via the Hypoxic Condition and Inflammation, Accompanied by Hypoxia-induced Autophagy Increased *In Vitro*

**Fig. 2:**
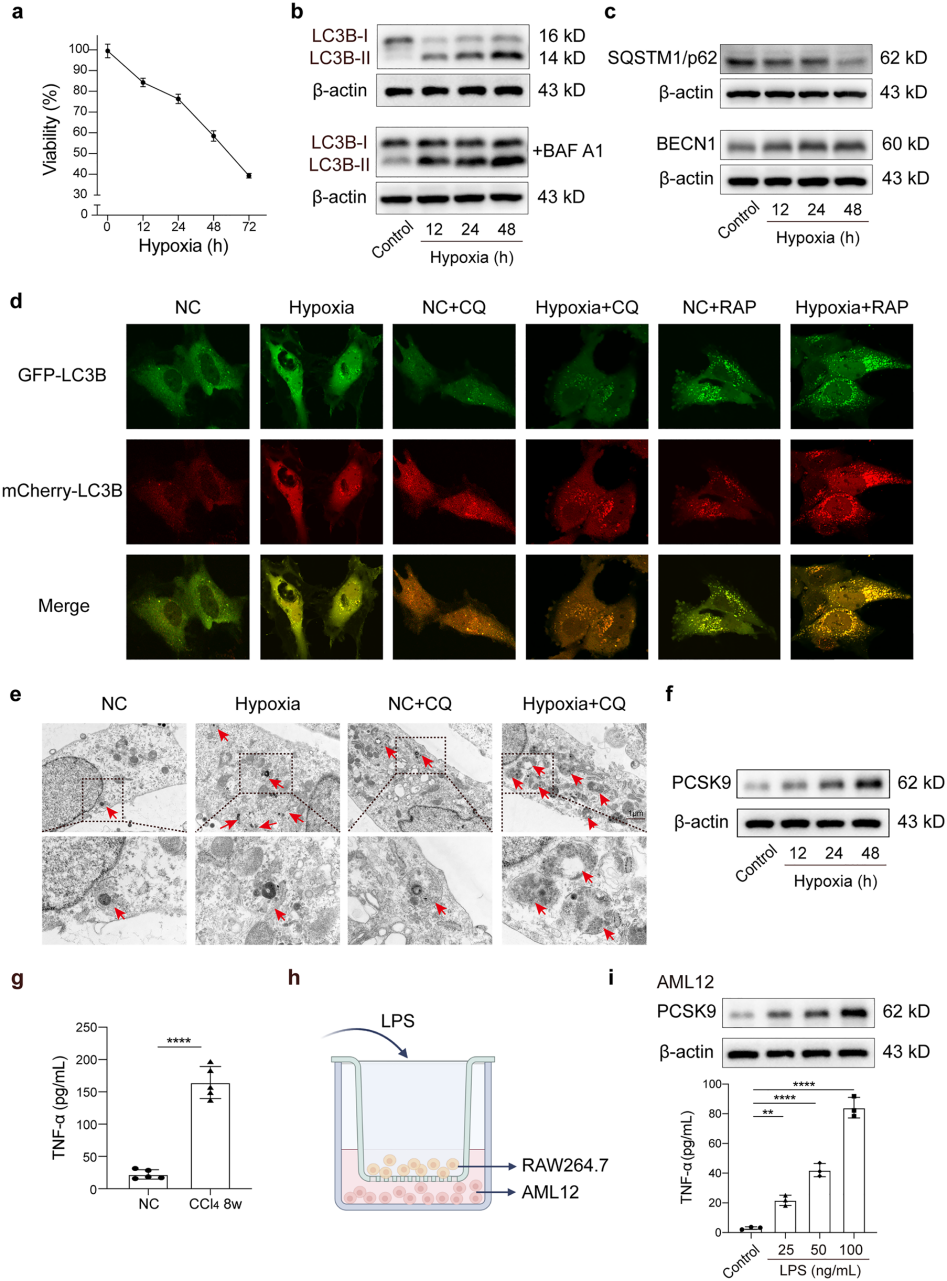
PCSK9 increased via the hypoxic condition and inflammation, accompanied by hypoxia-induced autophagy increased in vitro. **a** Cell viability assay of hepatocytes after exposure to the hypoxic condition for 0–72 h. **b**, **c** Hypoxia induced autophagic flux (LC3B, p62, and BECN1 expression). **d** Hepatocytes infected with Ad-mCherry-GFP-LC3B and  treated with hypoxia; autophagic flux detected using live-cell imaging microscopy following the administration of autophagy inhibitor chloroquine (CQ) or the inducer rapamycin (RAP). **e** TEM images of autophagosomes and autolysosomes in hepatocytes after hypoxia treatment. **f** Hypoxia induced PCSK9 expression. **g** ELISA of serum TNF-α levels in mice treated with CCl_4_ for 8 weeks (*n* = 5 mice/group). **h** Schematic diagram of co-cultured mouse hepatocyte line AML12 and mononuclear macrophage cell line RAW264.7. **i** PCSK9 expression in the hepatocytes and TNF-α level in the supernatant detected by ELISA after co-cultured AML12 and RAW264.7 treated with different concentrations of LPS (0–100 ng/mL); Western blot representative of multiple independent experiments (*n* = 3); data on serum TNF-α levels and cell viability from each experiment (*n* = 5) shown in graphs; data presented as mean ± standard deviation; multiple comparisons performed by one-way ANOVA; *p < 0.05; **p < 0.01; ***p < 0.001; ****p < 0.0001. Baf A1, bafilomycin A1.

Since hypoxia accompanies the whole process of liver fibrosis, we investigated the effect of hypoxia on hepatocytes. With the prolongation of hypoxic culture time, the viability of hepatocytes decreased gradually (Fig. 2a). Exposed to the hypoxic condition for 72 h, the cell viability was below 50%. Therefore, we set the maximum time of hypoxic treatment at 48 h in the subsequent experiments.

In view of the stress state of hypoxia that could induce autophagy [22], we further examined the autophagy in hepatocytes under the hypoxic condition, finding that it increased with prolonged hypoxia, as evidenced by the increased expression of LC3B-II (Fig. 2b) and BECN1, and the decreased expression of SQSTM1/p62 (Fig. 2c). To further confirm the effect of hypoxia on autophagic flux, the hepatocytes were infected with adenovirus expressing mCherry-GFP-LC3B fusion protein to measure autophagic flux based on the principle of GFP quenching in lysosomes following the fusion of autophagosomes and lysosomes. After the use of the autophagy inhibitor chloroquine (CQ) or the inducer rapamycin (RAP), the number of red and yellow spots increased after hypoxia treatment, indicating that hypoxia-induced autophagy was not altered by CQ or RAP when compared with the controls (Fig. 2d). As indicated by transmission electron microscope (TEM) images, hypoxia increased the number of autophagosomes and autolysosomes in hepatocytes, which were not affected by CQ (Fig. 2e).

With the evidence that PCSK9 expression increased during liver fibrosis, we examined the effect of hypoxia on PCSK9 expression in hepatocytes. As shown in Fig. 2f, PCSK9 expression was enhanced when the hypoxia treatment was prolonged, which indicated that the expression of PCSK9 in hepatocytes was regulated by hypoxia.

Admittedly, liver fibrosis is accompanied by increased inflammation; therefore, we investigated whether inflammation could affect the expression of PCSK9 in hepatocytes. The results showed that the chronic administration of CCl_4_ resulted in hepatic inflammation and fibrosis in the mice and that a significant increase was detected in serum TNF-α levels, one of the main injury factors of the liver (Fig. 2g). Subsequently, we stimulated the co-cultured mouse hepatic cell line AML12 and mononuclear macrophage cell line RAW264.7 with different concentrations of LPS (0–100 ng/mL) to simulate the inflammatory state in vivo (Fig. 2h). Consequently, the content of TNF-α was detected by ELISA in the supernatant. The results showed that as the LPS stimulation concentration increased, the content of TNF-α in the supernatant and the expression of PCSK9 in hepatocytes increased bit by bit (Fig. 2i).

Thus, the increased expression of PCSK9 was regulated by hypoxia and inflammation, accompanied by hypoxia-induced autophagy increased.

### The Bidirectional Relationship Between Hypoxia-ROS and PCSK9

**Fig. 3:**
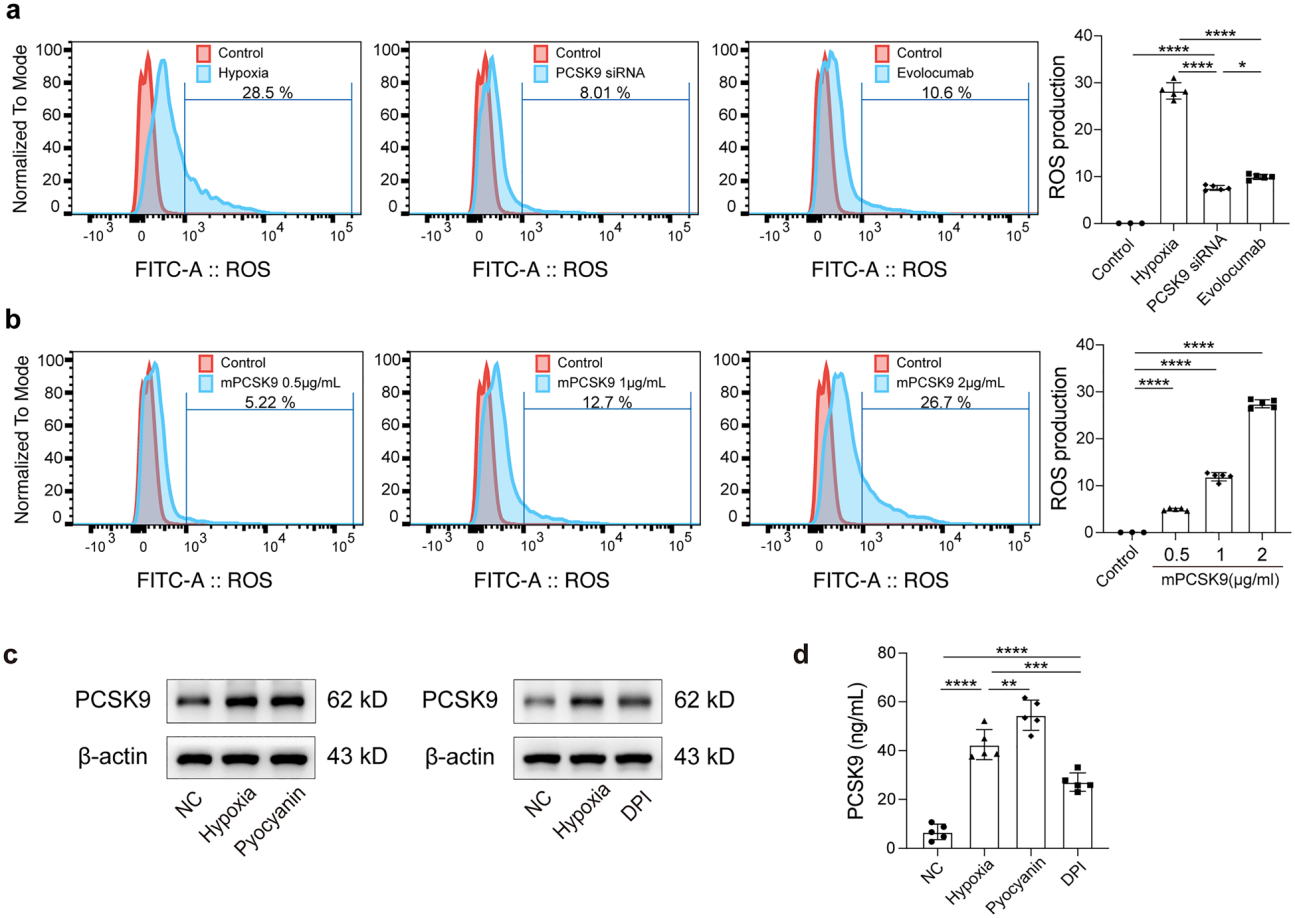
The bidirectional relationships of hypoxia-ROS and PCSK9. **a** Flow cytometry detection of ROS generation in the hepatocytes treated with hypoxia and PCSK9 inhibition (by PCSK9 siRNA and Evolocumab). **b** Flow cytometry detection of ROS generation in the hepatocytes after mPCSK9 protein stimulation. **c** Western blot of PCSK9 protein expression of the hepatocytes treated with the ROS inducer pyocyanin and the inhibitor diphenyleneiodonium chloride (DPI) under the hypoxic condition. **d** ELISA for PCSK9 levels in the supernatants of the hepatocytes exposed to hypoxia with the ROS inducer pyocyanin and the inhibitor DPI; Western blot representative of multiple independent experiments (*n* = 3); the in vitro data obtained from five independent experiments; data presented as mean ± standard deviation; the multiple comparisons performed by one-way ANOVA; *p < 0.05; **p < 0.01; ***p < 0.001; ****p < 0.0001.

Since ROS has been proven to be the main product of hypoxia through a great number of oxygen free radicals arising [23], we used ROS to assess the degree of hypoxia and further investigated the relationship between hypoxia-ROS and PCSK9. From this, the level of ROS was detected by flow cytometry. The results indicated that hypoxia induced ROS production, which was partially counteracted by the inhibition of PCSK9 with PCSK9 siRNA or Evolocumab (Fig. 3a). Furthermore, ROS was also detected when the hepatocytes were stimulated with recombinant mouse PCSK9 protein (mPCSK9, 0.5–2 μg/mL). The results showed that mPCSK9 also induced ROS generation in a dose-dependent manner (Fig. 3b). Additionally, the ROS inducer pyocyanin and the inhibitor diphenyleneiodonium chloride (DPI) were used to explore the effect of hypoxia-induced ROS production on PCSK9 expression. Western blot showed that the ROS inducer pyocyanin increased PCSK9 expression under the hypoxic condition, whereas the ROS inhibitor DPI inhibited PCSK9 expression (Fig. 3c). The involvement of hypoxia-induced ROS in PCSK9 regulation was further supported in ELISA analysis of PCSK9 secretion in hepatocyte supernatant (Fig. 3d). These findings suggested a bidirectional interaction between hypoxia-ROS and PCSK9.

### PCSK9-Raised Autophagy as Hypoxia in Primary Hepatocytes

**Fig. 4:**
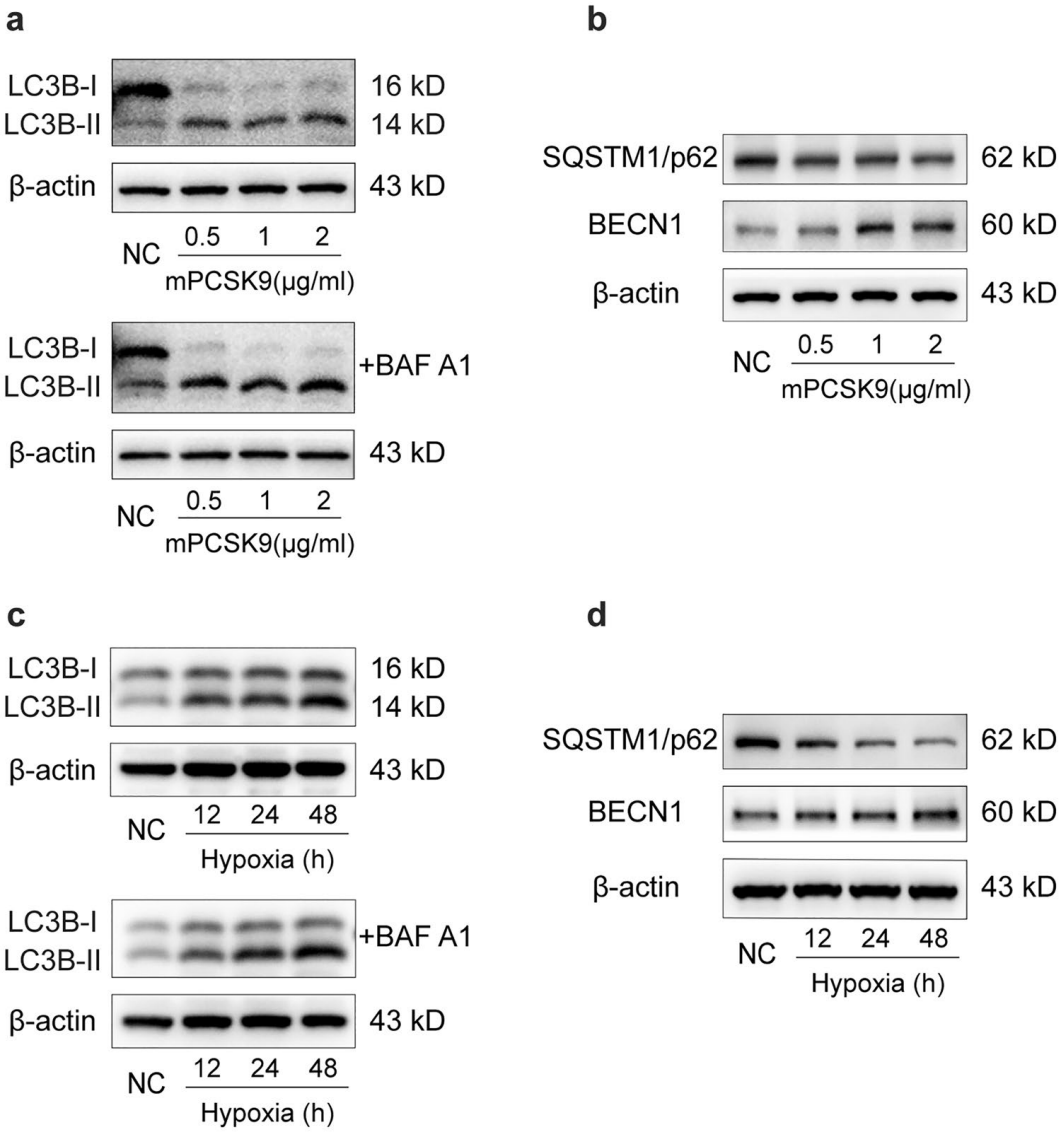
Relationship between PCSK9, hypoxia, and the development of autophagy in primary hepatocytes. **a** Western blot of LC3B protein expression in primary hepatocytes treated with mPCSK9 protein with/without BAF A1. **b** Western blot of SQSTM1/p62 and BECN1 protein expression in primary hepatocytes treated with mPCSK9 protein. **c** Western blot of LC3B protein expression in primary hepatocytes exposed to hypoxia with/without BAF A1. **d** Western blot of SQSTM1/p62 and BECN1 protein expression in primary hepatocytes exposed to hypoxia; Western blot representative of multiple independent experiments (*n* = 3); BAF A1, bafilomycin A1.

Based on the observations made, we further verified the relationship between PCSK9, hypoxia, and the development of autophagy in primary hepatocytes, which were treated with mPCSK9 protein (0.5–2 μg/mL) and the autophagic flux of which was measured. In practice, mPCSK9 dose-dependently raised the expression of LC3B-II (Fig. 4a) and BECN1 and decreased the expression of p62 (Fig. 4b). Furthermore, we examined the autophagic flux of primary hepatocytes under the hypoxic condition, finding that hypoxia also increased autophagy flux in primary hepatocytes, as indicated by LC3B-II (Fig. 4c) and BECN1 expression elevated, and p62 expression reduced (Fig. 4d).

### PCSK9 Expression and Autophagy Attenuated by Hypoxia Reversal

**Fig. 5:**
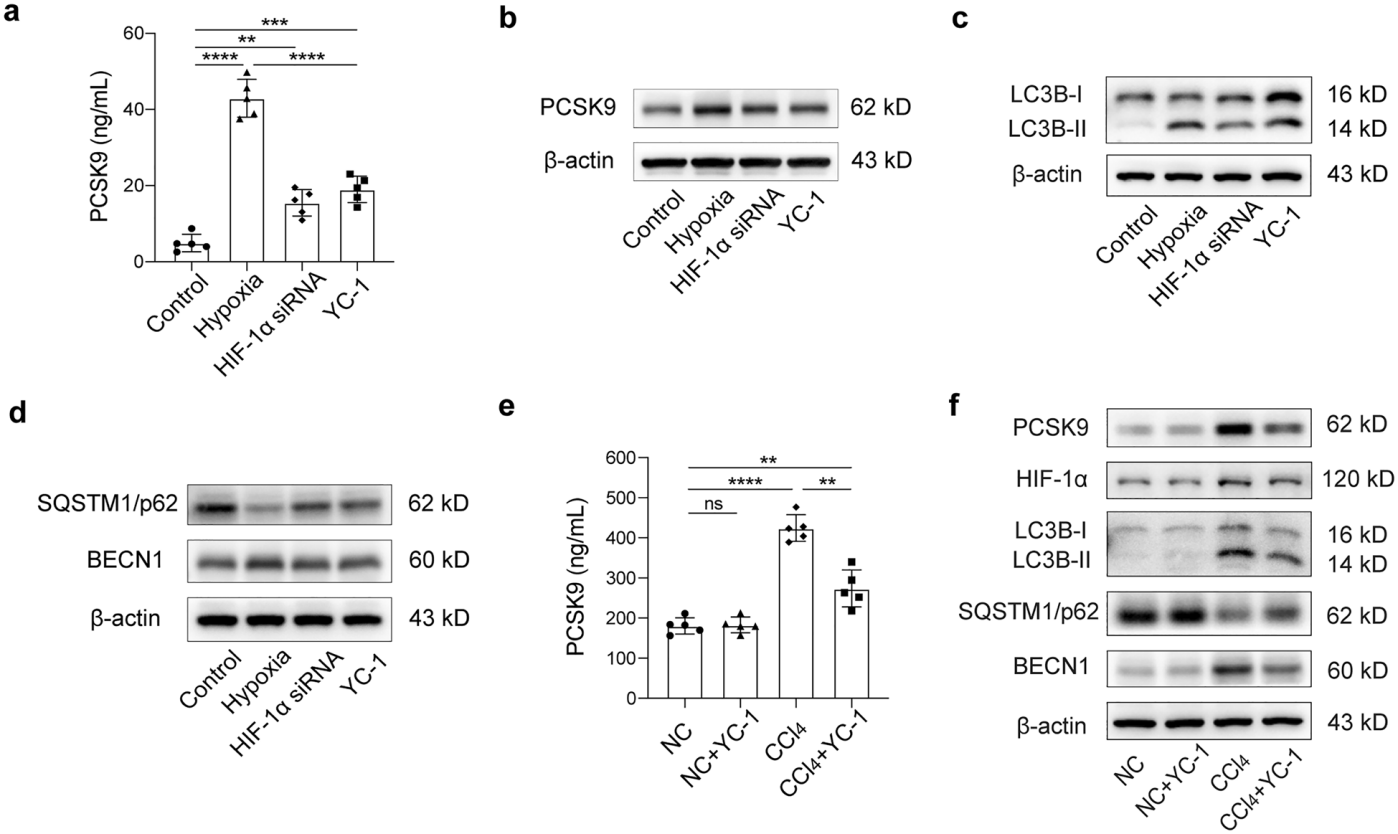
Role of HIF-1α in PCSK9 expression and autophagy. **a** ELISA for PCSK9 levels in the supernatant of hepatocytes exposed to hypoxia with/without HIF-1α inhibition (by using HIF-1α siRNA or YC-1). **b**–**d** Western blot of PCSK9, LC3B, SQSTM1/p62, and BECN1 expression in hepatocytes exposed to hypoxia for 24 h with/without HIF-1α siRNA or YC-1. **e** Serum PCSK9 levels in NC and CCl_4_-treated mice with/without HIF-1α inhibition (by using the chemical inhibitor YC-1). **f** Western blot of PCSK9 and autophagic flux in NC and CCl_4_-treated mice with/without YC-1; Western blot representative of multiple independent experiments (*n* = 3); in vivo studies conducted in the mice (*n* = 5 per group) and in vitro cell data obtained from several independent experiments (*n* = 5); data presented as mean ± standard deviation; multiple comparisons performed by one-way ANOVA; *p < 0.05; **p < 0.01; ***p < 0.001; ****p < 0.0001. NC, negative control.

HIF-1α is a critical regulator of cellular and systemic responses to low oxygen levels, and hepatocyte hypoxia contributes directly to the progression of liver fibrosis; therefore, we hypothesized that HIF-1α could play an important role in PCSK9 expression and autophagy during liver fibrosis. This hypothesis was examined in the hepatocytes with HIF-1α siRNA and YC-1, a chemical inhibitor of HIF-1α. In vitro experiments showed that the expression/release of PCSK9 was significantly increased with the hypoxia treatment by ELISA (Fig. 5a) and Western blot (Fig. 5b), which was significantly inhibited by the pretreatment of HIF-1α siRNA or YC-1. Meanwhile, the hypoxia treatment increased autophagic flux in the hepatocytes, as evidenced by the elevated expression of LC3B-II and BECN1 and decreased expression of p62. This tendency was significantly attenuated when the hepatocytes were transfected with HIF-1α siRNA or treated with YC-1 (Fig. 5c, d). In vivo experiments showed that the serum level of PCSK9 in CCl_4_-treated mice was significantly reduced after YC-1 administration (Fig. 5e). Western blot also showed that YC-1 significantly decreased the expression of PCSK9, LC3B-II, and BECN1, respectively, and increased the expression of p62 during liver fibrosis (Fig. 5f). Taken together, these data suggested that the reversal of hypoxia could reduce PCSK9 expression and autophagy both in vivo and in vitro.

### Liver Inflammation, Fibrosis, Hypoxia and Autophagy Attenuated by PCSK9 Inhibition in CCl_4_-Treated Mice

Given the central role of PCSK9 in mediating hypoxia-induced autophagy, we investigated its potential impact on CCl_4_-induced liver fibrosis in the mice. Since PCSK9 expression and autophagic flux reached the maximum 8 weeks after CCl_4_ treatment, the timepoint was chosen for the subsequent experiments. PCSK9-inhibited mice were generated in two ways: PCSK9 inhibited exogenously by weekly subcutaneous injection of Evolocumab and PCSK9 inhibited endogenously via the tail vein injection of CRISPR-Cas9 AAV 2 weeks before CCl_4_ treatment and again 1 month later.

**Fig. 6:**
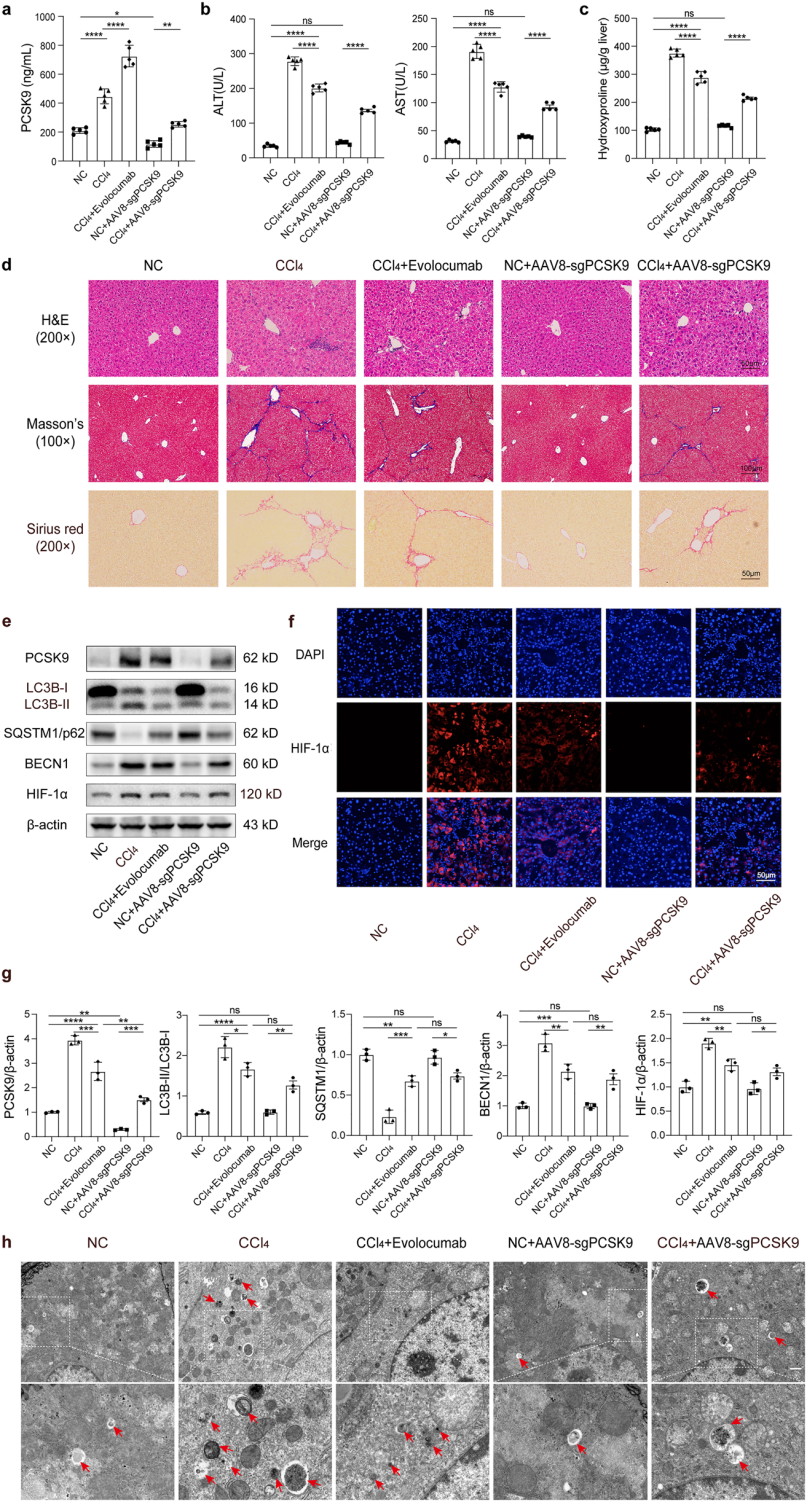
Hepatic function, PCSK9 expression, and autophagy in the liver of CCl_4_-treated mice pretreated with Evolocumab or AAV8-sgPCSK9. **a** Serum PCSK9 levels in the mice pretreated with PCSK9 inhibitor Evolocumab and AAV8-sgPCSK9. **b** Serum ALT and AST levels. **c** Hydroxyproline content in the liver tissue. **d** H&E, Masson’s, and Sirius red staining of the liver tissue sections. **e** Western blot of PCSK9, LC3B, SQSTM1/p62, BECN1, and HIF-1α protein expression in the liver tissue. **f** Immunofluorescence staining of HIF-1α in the liver sections. **g** Quantification of relative protein levels calibrated with β-actin. **h** TEM images of autophagosomes and autolysosomes in the liver (scale bar: 10 μm); in vivo studies conducted in the mice (*n* = 5 per group); Western blot representative of multiple independent experiments (*n* = 3); data presented as mean ± standard deviation; multiple comparisons performed by one-way ANOVA; *p < 0.05; **p < 0.01; ***p < 0.001; ****p < 0.0001.

As indicated in Fig. 6a, the serum PCSK9 level was detected in each group. Both the exogenous and endogenous inhibition of PCSK9 decreased serum ALT and AST levels in CCl_4_-treated mice (Fig. 6b). Similarly, H&E staining of the liver tissue sections showed that PCSK9 inhibition reduced the infiltration of inflammatory cells in the liver (Fig. 6d). In the mice pretreated with Evolocumab and AAV8-sgPCSK9, the extent of CCl_4_-induced pathological changes in hydroxyproline, Masson’s, and Sirius red staining of the damaged liver tissue sections was significantly reduced (Fig. 6c, d).

From the assessment of hypoxia marker HIF-1α and autophagic flux (LC3B, SQSTM1/p62, and BECN1), additionally, the results reconfirmed that PCSK9 expression was positively correlated with hypoxia-induced autophagy and that PCSK9 inhibition attenuated the degree of hypoxia and autophagy in the liver of CCl_4_-treated mice, as shown by the decreased expression of HIF-1α, LC3B-II, and BECN1 and the increased expression of p62 (Fig. 6e, g). Immunofluorescent staining of the liver tissue sections also showed that PCSK9 inhibition reduced HIF-1α expression in the fibrotic liver tissue (Fig. 6f), where TEM was used to observe the formation of autophagosomes and autolysosomes. As shown in Fig. 6h, the CCl_4_-treated group presented more autophagic vesicles, while the application of Evolocumab and AAV8-sgPCSK9 reduced the number of autophagosomes and autolysosomes.

These findings indicated that PCSK9 inhibition significantly ameliorated liver inflammation and fibrosis in the CCl_4_-treated mice, while attenuating hepatic hypoxia and autophagy.

### The Role of AMPK/mTOR/ULK1 Signaling Pathway in Hypoxia-Induced Autophagy Regulated by PCSK9 in Liver Fibrosis

**Fig. 7:**
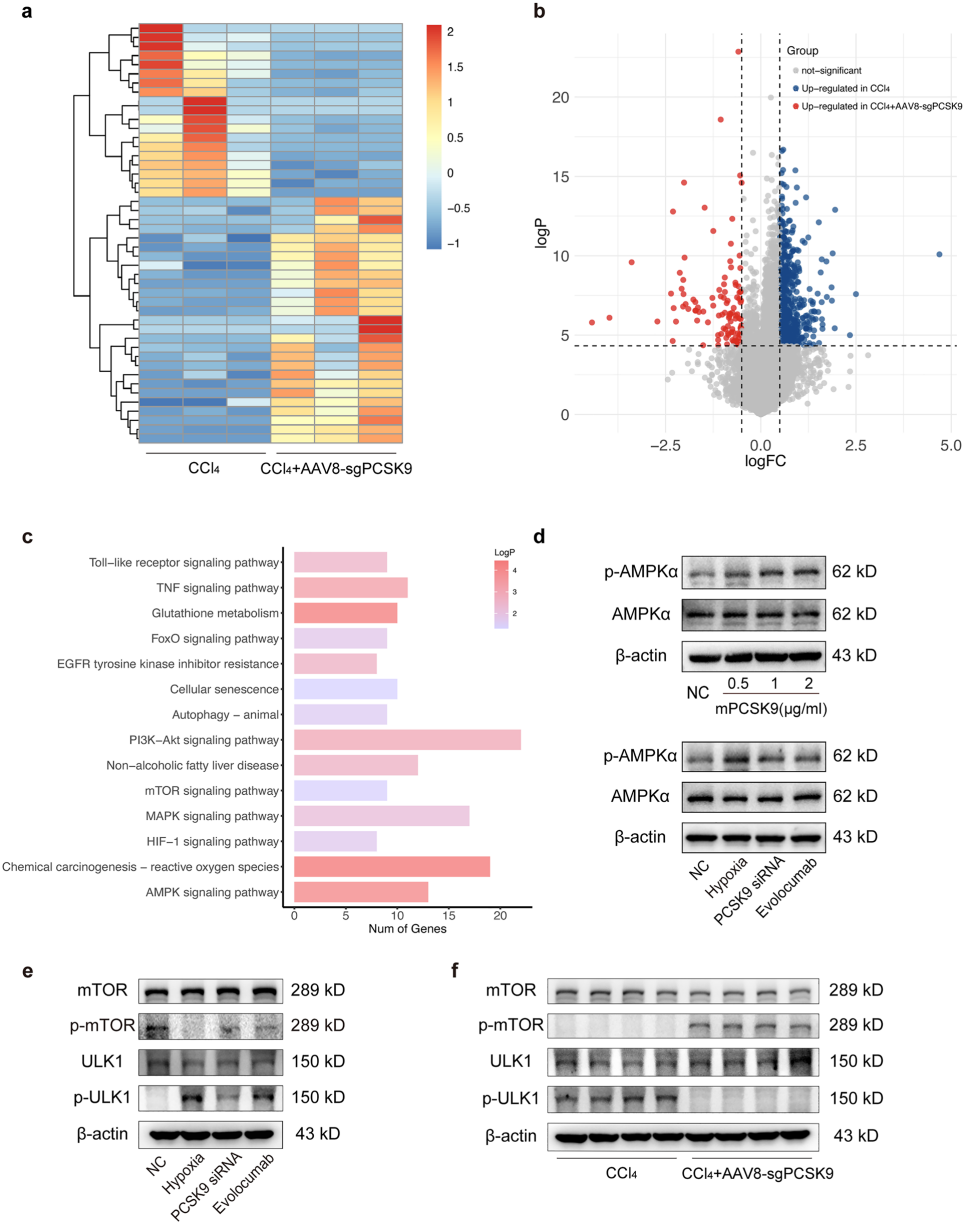
PCSK9-regulated hypoxia-induced autophagy through AMPK/mTOR/ULK1 signaling pathway. **a** The heat map showing differential genes of CCl_4_ and CCl_4_ + AAV8-sgPCSK9 mice. **b** The volcano map showing differential genes of CCl_4_ and CCl_4_ + AAV8-sgPCSK9 mice. **c** Enriched pathways of KEGG pathway analysis. **d** Western blot of AMPKα and p-AMPKα protein expression in the hepatocytes treated with mPCSK9 protein, hypoxia, and PCSK9 inhibition (by PCSK9 siRNA and Evolocumab). **e** Western blot of mTOR and ULK1 protein expression in the hepatocytes treated with hypoxia and PCSK9 inhibition (by PCSK9 siRNA and Evolocumab). **f** Western blot of mTOR and ULK1 protein expression in the fibrotic liver tissue of CCl_4_ and CCl_4_ + AAV8-sgPCSK9 mice; Western blot representative of multiple independent experiments (*n* = 3).

To elucidate the mechanism of PCSK9 regulating hypoxia-induced autophagy in liver fibrosis, we performed transcriptome sequencing of the liver tissues from CCl_4_-treated and CCl_4_ + AAV8-sgPCSK9 mice, as indicated by the heat map and volcano map of differential genes (Fig. 7a, b). KEGG pathway analysis indicated the significant enrichment of AMPK (adenosine 5′-monophosphate (AMP)-activated protein kinase) signaling pathway, oxidative stress, and autophagy pathways (Fig. 7c).

We further verified the effect of PCSK9 and hypoxia on AMPK expression in the hepatocytes, the outcomes demonstrating that mPCSK9 protein enhanced the expression of p-AMPKα dose-dependently and that hypoxia treatment raised the expression of p-AMPKα, but PCSK9 suppression using siRNA transfection or Evolocumab decreased the production of p-AMPKα, which suggested that PCSK9 and hypoxia were involved in the regulation of AMPK expression (Fig. 7d).

As the upstream key molecules of autophagy regulation, the expression of mTOR and ULK1 in the hepatocytes and liver tissues was further detected. According to Western blot analysis, hypoxia treatment reduced the expression of p-mTOR and enhanced the expression of p-ULK1 significantly in the hepatocytes, whereas PCSK9 inhibition reversed partially the hypoxia-induced changes in the expression of p-mTOR and p-ULK1 (Fig. 7e). Likewise, PCSK9 inhibition raised the expression of p-mTOR and decreased the expression of p-ULK1 significantly in the fibrotic liver tissues of the CCl_4_-treated mice (Fig. 7f).

**Fig. 8:**
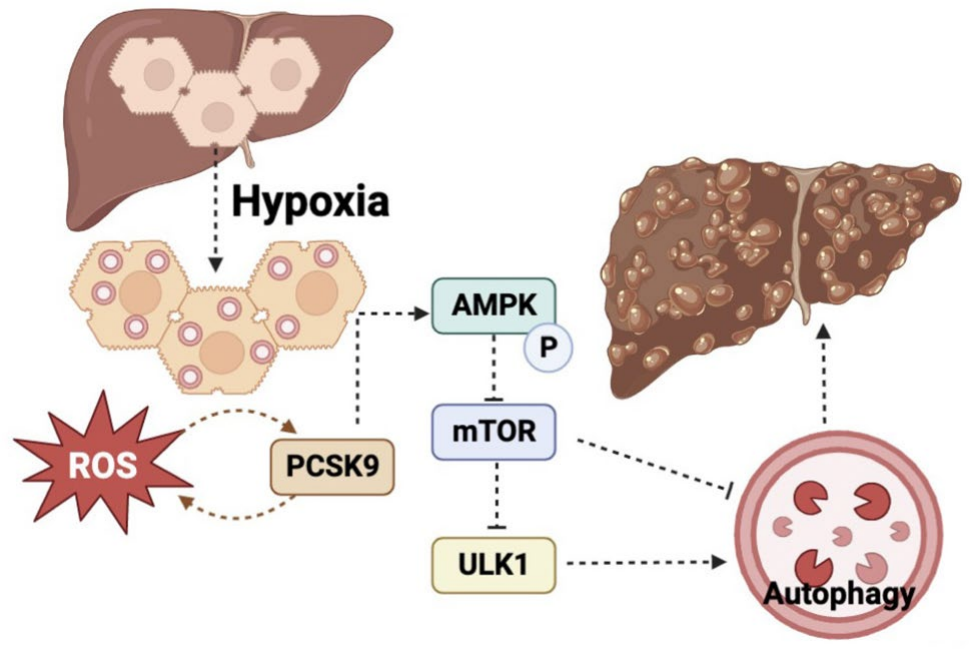
Mechanistic diagram of PCSK9 mediating hypoxia-induced autophagy in the hepatocytes through the AMPK/mTOR/ULK1 signaling pathway during liver fibrosis.

Overall, the findings indicated that anti-PCSK9 treatment improved liver fibrosis by regulating hypoxia-induced autophagy in hepatocytes through the AMPK/mTOR/ULK1 signaling pathway (Fig. 8).

## DISCUSSION

Anti-PCSK9 therapies have gained prominence in the treatment of hypercholesterolemia, and no serious adverse events have been observed from laboratory to clinical application. At present, however, the data were obscure when the side effect of anti-PCSK9 therapy is considered on liver function, especially when hepatocytes are the main cells producing PCSK9, and cholesterol metabolism is also mainly completed by hepatocytes [24]. It is of great importance for us to investigate the effect of PCSK9 on hepatic metabolism and biological function.

A previous investigation of mouse plasma, liver, and bile acids revealed that PCSK9-deficient mice had well-maintained cholesterol metabolism and hepatic homeostasis [25]. PCSK9 mAb therapy significantly reduced hepatic steatosis, liver inflammation, and fibrosis in patients with severe hyperlipidemia [26, 27]. It appears that anti-PCSK9 therapy not only improves hypercholesterolemia but also treats steatohepatitis. However, some studies have found the unfavorable evidence that high-cholesterol diets in PCSK9 knockout mice showed increasing hepatic free cholesterol and cholesterol crystals, and fibrotic steatohepatitis, even with a higher predisposition to liver cancer as compared with WT mice [28], and that PCSK9 KO exacerbated NASH, fibrosis, and liver injury in the presence of excess dietary fats in mice [13]. These findings suggested that anti-PCSK9 could not protect against hepatic steatosis and liver injury.

In view of which, we are not in a position to know whether anti-PCSK9 treatment can improve liver fibrosis. As previously reported, we verified that PCSK9 expression was elevated in the mice and human fibrotic liver samples, and PCSK9 inhibition by curcumin or PCSK9 KO significantly alleviated liver inflammation and fibrosis [11, 17]. In the current study, we used a CCl_4_-induced model of canonical liver fibrosis mice to get a deep insight into the mechanism, with the application of both exogenous PCSK9 inhibition with PCSK9 mAb Evolocumab and endogenous PCSK9 inhibition by CRISPR-Cas9 AAV.

It is well recognized that PCSK9 acts as a cholesterol regulator and a putative cytokine with pro-inflammatory effect. It was reported that the mRNA levels of IL-1, IL-6, TNF-α, CXCL2, and CCL2 increased when macrophages were incubated with recombinant or HepG2-derived PCSK9 protein [29]. PCSK9 inhibition-caused overexpression of hepatic LDLR promoted LPS clearance, lowering the inflammatory response and increasing survival time in the mice after sepsis [9, 10]. In the patients with PCSK9 loss-of-function mutations, better clinical outcomes were observed during septic shock [8]. In our previous study, similarly, PCSK9 inhibition was observed to reduce intestinal endotoxemia, thereby attenuating liver fibrosis [11].

In the current study, of note were the increase of PCSK9 that paralleled autophagy in the hepatocytes, and the inhibition of PCSK9 that decreased autophagy and alleviated liver fibrosis, which was different from any previously reported evidence. It is generally believed that autophagy in hepatocytes, endothelial cells, and macrophages indirectly protects against liver fibrosis, while autophagy in HSCs aggravates liver fibrosis [30]. Contrary to the widely accepted theory, however, our study demonstrated that PCSK9 inhibition reduced autophagy in the hepatocytes, thereby attenuating liver inflammation and fibrosis. Similar to our findings, the inhibition of hepatocyte autophagy was reported to ameliorate Fas/FasL-regulated hepatocyte apoptosis, HSC activation, and liver fibrosis [31]. For this, a plausible explanation is that the occurrence of autophagy is a dynamic process, which may be beneficial at the initial stage and harmful at an advanced stage [3], where autophagy could even lead to cell death [32, 33]. Hepatocytes were revealed to own a high level of autophagic flux due to the increased abundance of lysosomes and lysosomal enzymes, and enhanced autophagy could regulate the progression of hepatocyte death [34]. Therefore, enhanced autophagy is detrimental in hepatocytes, which further supports our findings.

Hypoxia, a common characteristic during the whole process of liver inflammation and fibrosis, affects mitochondrial respiratory chain function in hepatocytes, which in turn may increase intracellular ROS generation through the electron transport chain, an event that may lead to oxidative stress, cell damage, and death [35]. Studies have proved that hypoxia induces the expression of PCSK9 in cultured cardiomyocytes [36, 37]; on the other hand, PCSK9 induces mitochondrial dysfunction and ROS generation in endothelial cells in the context of atherosclerosis [38]. In our study, we found that hypoxia and its generated ROS induced the elevated expression of PCSK9 in the hepatocytes; that PCSK9 inhibition reduced hypoxia-induced ROS production; and that interfering with ROS production also affected the expression of PCSK9 under the hypoxic condition. From our study, both in vitro experiments and RNA-Seq analysis confirmed the evidence that a bidirectional interaction exists between PCSK9 and hypoxia-mediated ROS generation.

Autophagy is recognized as a common mechanism of modulating diverse signaling pathways [39]. A notable regulator of autophagy is the AMPK signaling pathway, which is activated by hypoxia and metabolic stress [40]. Metformin was reported to induce autophagy through the AMPK signaling pathway, relieve hypoxic stress, and promote the survival of random skin flaps by increasing neovascularization [41]. AMPK was indicated to be required for hypoxia-mediated autophagy, which protected cardiomyocytes from ischemia–reperfusion injury [42]. It is well recognized that AMPK increases autophagy effectively through multiple mechanisms. AMPK has been reported to repress the synthesis of mTOR complex 1 (mTORC1) through phosphorylating the TSC1-TSC2 complex, thereby reducing the activity of mTOR and alleviating the inhibition of mTOR on autophagy [43, 44]. As an evolutionarily conserved protein kinase, mTOR plays a negative regulatory role in autophagy by inhibiting ULK1 activation [45]. ULK1 was also phosphorylated and activated by AMPK, leading to the initiation of the autophagic cascade [46]. In the current study, we observed that PCSK9 promoted the phosphorylation and activation of AMPK, thereby reducing the activity of mTOR, promoting the activation of ULK1, and ultimately enhancing autophagy.

Additionally, it is of more importance to investigate serum PCSK9 levels than hepatic PCSK9 expression. A previously reported multivariable linear regression analysis indicated that non-alcoholic fatty liver disease (NAFLD) score levels were independently related to higher PCSK9 levels [47]. A further proof was provided that circulating PCSK9 concentrations were not associated with the severity of liver steatosis or histological markers of NASH [15]. Furthermore, those who had liver cirrhosis produced significantly lower serum PCSK9 concentrations [48], and those who had chronic hepatitis or liver cirrhosis showed serum PCSK9 levels that were 20–30% lower than the healthy controls [49]. In order to confirm the reflection effect of PCSK9 on liver biochemical function, further investigations need to be conducted.

In summary, our study demonstrates that the expression of PCSK9 is closely associated with the level of hypoxia-induced autophagy during the development of CCl_4_-induced hepatic fibrosis. Anti-PCSK9 treatment alleviates liver fibrosis by regulating hypoxia-induced autophagy in the hepatocytes through AMPK/mTOR/ULK1 signaling pathway. Thus, PCSK9 has promising potential of becoming a new biomarker as a therapeutic target for the treatment of hepatic fibrosis.

### Supplementary Information

Below is the link to the electronic supplementary material.

Supplementary file1 (DOCX 5755 KB)

## Data Availability

The data that support the findings of this study are available on request from the corresponding authors.

## Abbreviations

AAV: Adeno-associated virus
ALT: Alanine transaminase
AMPK: Adenosine 5′-monophosphate-activated protein kinase
AST: Aspartate transaminase
Baf A1: Bafilomycin A1
BSA: Bovine serum albumin
CCl_4_: Carbon tetrachloride
CQ: Chloroquine
DEGs: Differentially expressed genes
DPI: Diphenyleneiodonium chloride
GO: Gene Ontology
HIF-1α: Hypoxia-inducible factor-1α
HSCs: Hepatic stellate cells
IHC: Immunohistochemistry
KEGG: Kyoto Encyclopedia of Genes and Genomes
LDLR: Low-density lipoprotein receptor
LPS: Lipopolysaccharide
mAb: Monoclonal antibody
mTOR: Mammalian rapamycin targeting protein
NAFLD: Non-alcoholic fatty liver disease
NASH: Nonalcoholic steatohepatitis
PCSK9: Proprotein convertase subtilisin/kexin type 9
RAP: Rapamycin
ROS: Reactive oxygen species
siRNA: Small interfering RNA
TEM: Transmission electron microscope
TNF-α: Tumor necrosis factor-α
