# Supplementary material for: Anti-PCSK9 Treatment Attenuates Liver Fibrosis via Inhibiting Hypoxia-Induced Autophagy in Hepatocytes
Source: Inflammation. 2023 Jul 19;46(6):2102–19. doi: 10.1007/s10753-023-01865-8 (PMC10673768; doi:10.1007/s10753-023-01865-8)
Supplement: Supplementary file 1 — Supplementary file1 (DOCX 5755 KB) [file 10753_2023_1865_MOESM1_ESM.docx]

**Supplementary materials**

**Table S1.** **Reagents**

| **Reagent** | **Manufacturer** |
| --- | --- |
| Carbon tetrachloride (CCl_4_) | Macklin Inc., Shanghai, China |
| Evolocumab | Amgen Inc., USA |
| Albumin BovineⅤ | Beijing Solarbio Science & Technology Co., Ltd., Beijing, China |
| DMEM/F-12 medium | Zhong Qiao Xin Zhou Biotechnology Co., Ltd., Shanghai, China |
| DMEM medium | GenomBiotechnology Co., Ltd, Shanghai, China |
| RPMI 1640 medium | Genom Biotechnology |
| Fetal bovine serum | YOBBIO Co., Ltd., Shanghai, China |
| Penicillin and streptomycin | Genom Biotechnology |
| Trypsin-ethylenediaminetetraacetic acid | Genom Biotechnology |
| Lipo8000 Transfection Reagent | Beyotime Co., Ltd, Shanghai, China |
| Pcsk9 siRNA | GenoPharma Co., Ltd. Shanghai, China |
| Hif1a siRNA | GenoPharma |
| PCSK9 CRISPR-Cas9 adeno-associated virus (AAV) | Hanyin Biotechnology Co., Ltd, Shanghai, China |
| Lificiguat (YC-1) | MedChemExpress, Shanghai, China |
| Mouse PCSK9 Quantikine ELISA kit | R&D |
| Mouse TNF-α ELISA kit | Boster Biological Technology |
| RIPA | Beyotime |
| Cell Counting Kit-8 | Beyotime |
| BCA Protein Assay Kit | Beyotime |
| Bovine serum albumin | Sigma |
| Reactive Oxygen Species Assay Kit | Beyotime |
| Goat anti-rabbit or goat anti-mouse secondary antibodies | Jackson Immunoresearch Inc., USA |
| ECLTM Western Blotting Detection Reagents | EpiZyme, Shanghai, China |
| Cy3 conjugated Goat Anti-Rabbit IgG | Servicebio, Wuhan, China |
| Trizol reagent | Takara, Shiga, Japan |
| Rapamycin | MedChemExpress |
| Chloroquine | MedChemExpress |
| Pyocyanin | MedChemExpress |
| Diphenyleneiodonium chloride (DPI) | MedChemExpress |
| mouse PCSK9 protein | MedChemExpress |
| Ad-mCherry-GFP-LC3B | Beyotime |
| TruSeq Stranded mRNA Sample Prep Kit | Illumina, San Diego, CA |

**Table S2.** **RNA interference oligo**

| **Gene** | **sense（5'-3'）** | **antisense（5'-3'）** |
| --- | --- | --- |
| Pcsk9 siRNA1 | GCCUGGAGUUUAUUCGGAATT | UUCCGAAUAAACUCCAGGCTT |
| Pcsk9 siRNA2 | GGAAGUGGAAGACCUUAGUTT | ACUAAGGUCUUCCACUUCCTT |
| Pcsk9 siRNA3 | GCUACAGAUUGAACAAACUTT | AGUUUGUUCAAUCUGUAGCTT |
| Hif1a siRNA1 | GAGCUUUGGAUCAAGUUAATT | UUAACUUGAUCCAAAGCUCTT |
| Hif1a siRNA2 | GCUGACCAGUUACGAUUGUTT | ACAAUCGUAACUGGUCAGCTT |
| Hif1a siRNA3 | CCACCACUGAUGAAUCAAATT | UUUGAUUCAUCAGUGGUGGTT |

**Table S3. Primers for qRT-PCR analysis**

| **Gene** |  | **Sequence (5’-3’)** |
| --- | --- | --- |
| Pcsk9 | F | GAGACCCAGAGGCTACAGATT |
| Pcsk9 | R | AATGTACTCCACATGGGGCAA |
| Hif1a | F | ACCTTCATCGGAAACTCCAAAG |
| Hif1a | R | ACTGTTAGGCTCAGGTGAACT |
| β-Actin | F | TGACGTGGACATCCGCAAAG |
| β-Actin | R | CTGGAAGGTGGACAGCGAGG |

**Table S4.** **Primary antibodies for Western blot assay**

| **Antibody** | **Cat No.** | **Manufacturer** |
| --- | --- | --- |
| PCSK9 | ab31762 | Abcam |
| LC3B | T55992 | Abmart |
| SQSTM1/p62 | AF5312 | Beyotime |
| Beclin1/BECN1 | A7353 | ABclonal |
| HIF-1α | 36169 | CST |
| phospho-AMPKα | 2535 | CST |
| AMPKα | 5832 | CST |
| phospho-ULK1 | 6888 | CST |
| ULK1 | 8054 | CST |
| phospho-mTOR | 2974 | CST |
| mTOR | 2983 | CST |
| β-actin | P30002 | Abmart |

**Fig. S1.** **qRT-PCR and Western Blot of RNA interference**

**
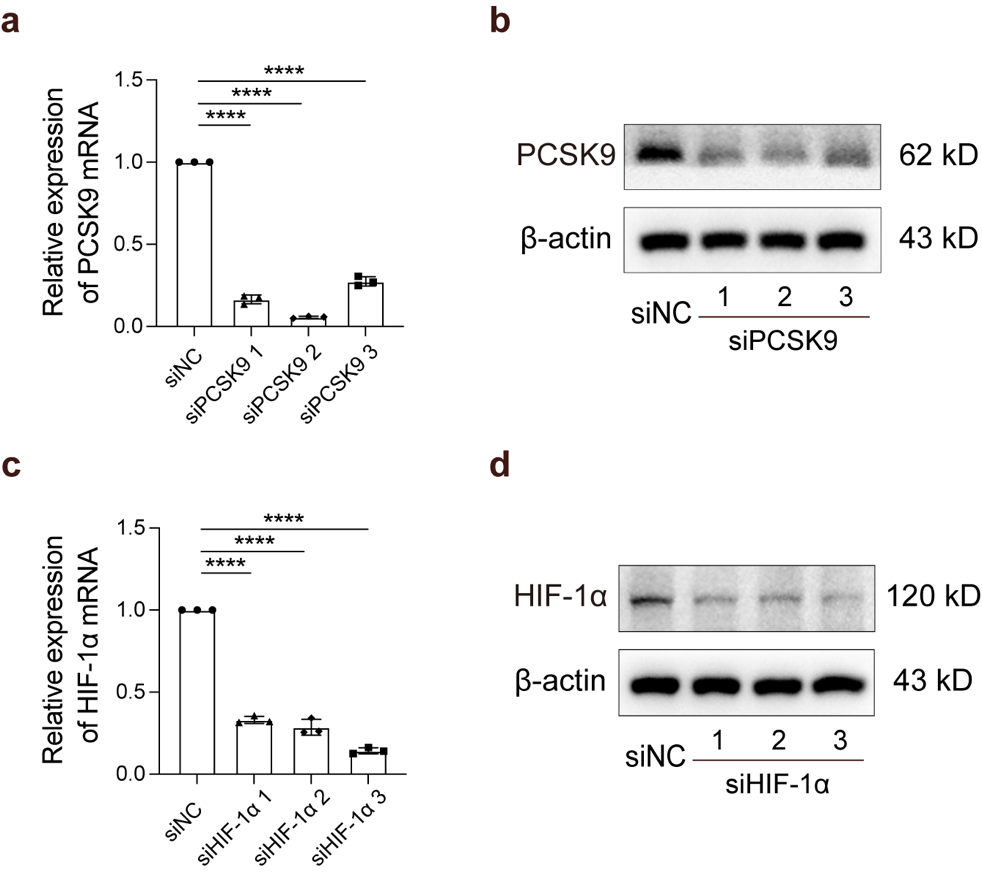
**

**Fig. S1** qRT-PCR and Western blot of RNA interference in hepatocytes. **a** qRT-PCR of PCSK9 mRNA expression in hepatocytes after transfected with PCSK9 siRNA. **b** Western blot of PCSK9 protein expression in hepatocytes after transfected with PCSK9 siRNA. **c** qRT-PCR of HIF-1α mRNA expression in hepatocytes after transfected with HIF-1α siRNA. **d** Western blot of HIF-1α protein expression in hepatocytes after transfected with HIF-1α siRNA; multiple comparisons performed by one-way ANOVA; data presented as mean ± standard deviation (n = 3/group); *****p* < 0.0001

**Fig. S2. Western blot of PCSK9 expression in other mouse tissues affected by AAV8-sgPCSK9.**


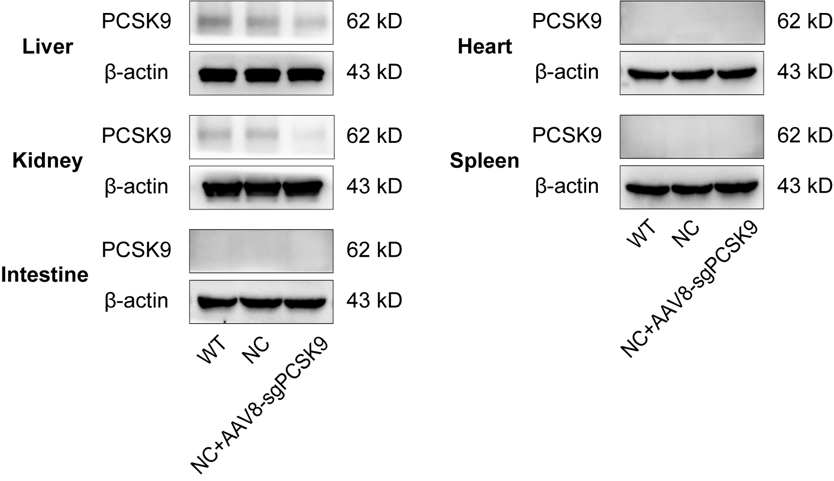


**Fig. S2** Western blot of PCSK9 expression in other mouse tissues affected by AAV8-sgPCSK9. WT, wild type; NC, negative control

**Fig. S3. Western blot of PCSK9 expression with two different antibodies.**


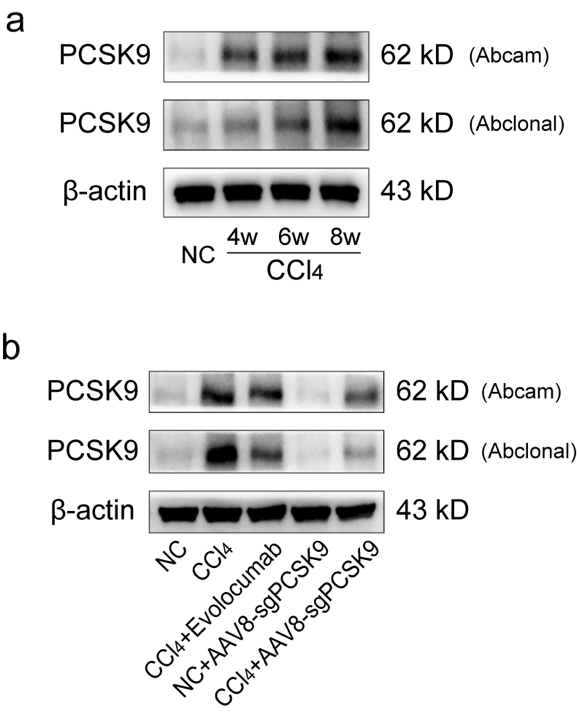


**Fig. S3** Western blot of PCSK9 expression with two different antibodies. **a** Western blot of PCSK9 expression in the mouse liver after CCl_4_ treatment; **b** Western blot of PCSK9 expression in the mouse liver after the treatment of Evolocumab or AAV8-sgPCSK9; the antibody in the top band as Abcam (ab1762); the antibody in the middle band as Abclonal (A21909); NC, negative control; CCl_4_, carbon tetrachloride

**Fig. S4. Immunohistochemical staining of PCSK9 protein in the liver of CCl_4_-treated mice.**

**
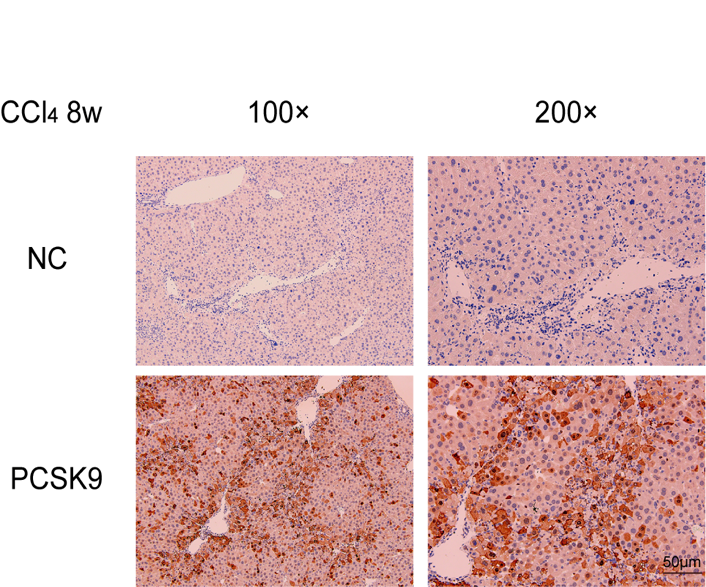
**

**Fig. S4** Immunohistochemical staining of PCSK9 protein in the liver of CCl_4_-treated mice. The negative control (NC) group without anti-PCSK9 antibody (ab1762) set to verify the specificity of the antibody
